# Nascent CUT&Tag captures transcription factor binding after chromatin duplication

**DOI:** 10.1101/2025.10.13.682212

**Published:** 2025-10-15

**Authors:** Matthew Wooten, Kevin Nguyen, Brittany N. Takushi, Kami Ahmad, Steven Henikoff

## Abstract

DNA replication strips off all chromatin proteins, which must be reassembled behind the replication fork. To track chromatin reassembly on newly synthesized DNA, we developed Nascent CUT&Tag, a chromatin profiling method that uses antibody-targeted *in situ* tagmentation to directly measure transcription factor binding on nascent chromatin. Using Nascent CUT&Tag, we tracked the recovery of GAGA factor (GAF) in Drosophila Kc cells. We find that GAF is displaced from chromatin during DNA replication and shows a broad spectrum of recovery times, ranging from minutes to hours. Early recovering peaks are characterized by shorter GAF motifs and are associated with functions related to cell cycle progression. Conversely, late recovering peaks are characterized by longer, degenerate GAF motifs and are associated with developmental functions. We also show that GAF recovery on newly synthesized DNA requires chromatin remodeling by Brahma Associated Factor (BAF), implying that nucleosome eviction is critical to fully reestablish GAF binding.

## Introduction

The majority of DNA in eukaryotic genomes is organized into nucleosomes, which are comprised of 146 base pairs of DNA wrapped around the histone octamer^1^. Transcription factors (TFs) are DNA binding proteins that alter chromatin structure and regulate gene expression by binding to targeted DNA sequences^2,3^. Nucleosome occupancy antagonizes TF binding, as most TFs cannot recognize and bind the target motifs wrapped around nucleosomes. DNA replication poses a formidable challenge to TF binding, as TFs become displaced from DNA following passage of the replication fork and replaced by nucleosomes^4,5^. To reestablish parental chromatin structure, TFs must overcome nucleosome occlusion to access and bind to their target motifs. However, despite the critical role that TFs play in regulating chromatin structure and gene expression, little is known regarding the kinetics or mechanisms by which TFs reassociate with DNA following passage of the replication fork.

To directly track the return of chromatin features to newly synthesized DNA, we developed a chromatin profiling method called Nascent CUT&Tag, which uses targeted *in* situ tagmentation to directly measure transcription factor binding on EdU-labeled nascent chromatin^6^. We used Nascent CUT&Tag to track the return of GAGA factor (GAF) in *Drosophila melanogaster* Kc167 cells. GAF is an essential *Drosophila* zinc finger TF encoded by the Trithorax-like (*Trl*) gene^7^ that binds to GA-rich sequence arrays on the promoters and cis-regulatory elements^8^ to drive gene activation and repression^9,10^. Using Nascent CUT&Tag, we find that GAF is displaced from chromatin during DNA replication and shows a range of recovery kinetics. We find a subset of GAF sites that recover binding minutes after fork passage, while other sites recover binding hours later. Early recovering GAF peaks contain short GAF DNA motifs and are located at genomic features associated with cell cycle progression. Conversely, late recovering GAF sites are characterized by longer, more degenerate GAF motifs and are found at genomic features associated with developmental functions. Using the small molecule inhibitor BRM014, we show that BAF activity is essential for GAF to fully recover binding to nascent chromatin. Together, this work reveals a role for nucleosome remodeling in facilitating TF binding to newly synthesized DNA, and suggests that motif structure plays a role in GAF recovery on nascent chromatin.

## Results

### Nascent CUT&Tag profiles chromatin features on newly synthesized DNA

**Figure 1.**
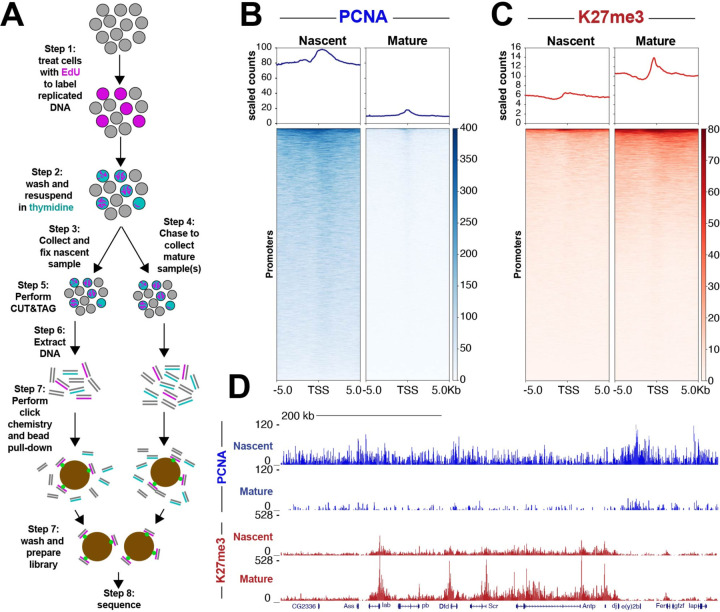
Nascent CUT&Tag captures changes in PCNA and H3K27me3 behind the replication fork (**A**) Experimental design for Nascent CUT&Tag workflow. (**B**) Heatmap aligned to transcription start site (TSS) of all promoters showing H3K27me3 counts scaled by number of mapped reads. (norm.). (**C**) Heatmap aligned to transcription start site (TSS) of all promoters showing PCNA counts scaled by number of mapped reads. (**D**) Representative UCSC browser tracks snapshot of PCNA and H3K27me3. Merged data of three biological replicates for PCNA and H3K27me3.

To detect factor binding on nascent DNA, we combined pulse-labeling of newly synthesized DNA with Cleavage Under Targets and Tagmentation (CUT&Tag) chromatin profiling (Figure 1A). We pulse-label Kc167 cells with a 15-minute treatment of 5-Ethynyl-2’-deoxyuridine (EdU), followed by increasing chase times to capture factor binding at different stages of chromatin maturation. Cells are then lightly fixed and processed for CUT&Tag with antibodies to a chromatin factor. Following tagmentation, we use click chemistry to link a biotin moiety to incorporated EdU, and then streptavidin pulldown to purify replicated DNA. PCR with tagmentation primers thus generates sequencing libraries specifically from replicated chromatin. Libraries were pooled by equal volumes to accurately represent amounts of chromatin-bound factors between timepoints.

To validate Nascent CUT&Tag, we first tested if we could detect the replication-coupled processivity factor PCNA on new DNA, as this should be specifically enriched in nascent libraries^11,12^. Indeed, we observe strong PCNA signal on newly synthesized DNA, while more mature chromatin shows reduced signal (Figure 1B,D). To further validate Nascent CUT&Tag, we measured the maturation kinetics for trimethylation at the histone H3 K27 residue (H3K27me3). This histone modification is catalyzed by the Polycomb repressive complex (PRC2) and promotes gene repression. Levels of H3K27me3 are initially diluted by half following passage of the replication fork, as duplicated chromatin contains an approximately equal mixture of old histones with the H3K27me3 mark and new histones that are unmarked. Over the course of hours, PRC2 methylates new histones, leading to a 2-fold increase in K27me3 levels from nascent chromatin to mature^13,14^. Indeed, H3K27me3-targeted Nascent CUT&Tag signals are low on newly replicated chromatin and then increase approximately two-fold on mature chromatin (Figure 1C,D). These experiments demonstrate that Nascent CUT&Tag quantitatively profiles diverse chromatin features on newly synthesized DNA.

### Transcription factors rapidly return to nascent chromatin

Nascent chromatin must mature to reestablish nucleosome organization and transcription factor binding. To describe the kinetics, we chose to profile GAF, an abundant zinc-finger transcription factor that is crucial for developmental gene expression programs in *Drosophila*. GAF binds at thousands of gene promoters, developmental enhancers, as well as heterochromatic satellite blocks that have AAGAG and other GA-rich motifs^10,15^. From bulk profiling, we annotated ~7,000 sites throughout the genome of *Drosophila* Kc167 cells (Supplementary Table 1). To then assess GAF binding to nascent DNA, we pulsed-labeled cells with EdU and then immediately prepared cells for profiling (T0), or chased cultures with thymidine for 1 or 4 hours (T1 and T4). These timepoints capture newly synthesized chromatin and progressively more mature chromatin after replication.

**Figure 2.**
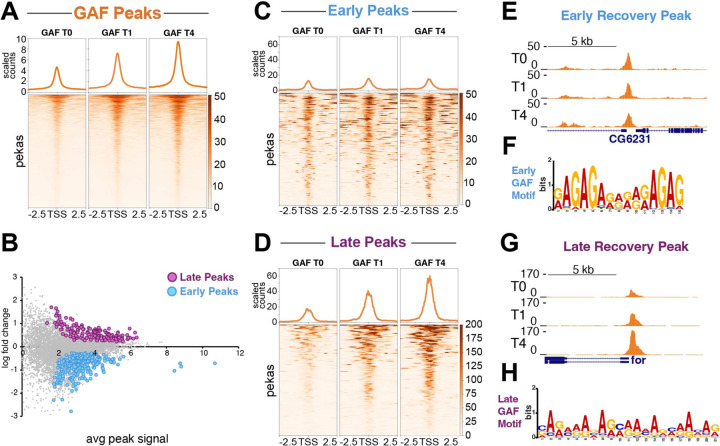
GAF binding is lost on nascent chromatin and requires BAF to recover (**A**) Heatmap aligned to the center of all GAF peaks showing GAF signal over the course of Nascent CUT&Tag experiment. T0 is newly synthesized chromatin immediately after replication fork passage. T1 is one hour after replication fork passage and T4 is four hours after replication fork passage. Read counts are scaled by number of mapped reads. (**B**) MA plot showing fold change in normalized counts comparing T4 to T0. Statistical significance determined by two-sided t-test. (**C**) Heatmap aligned to the center of all early recovering GAF peaks showing GAF signal over the course of Nascent CUT&Tag experiment (**D**) Heatmap aligned to the center of late recovering GAF peaks showing GAF signal over the course of Nascent CUT&Tag experiment. (**E**) Representative UCSC browser track snapshot centered on the promoter of CG6231 showing GAF signal across Nascent CUT&Tag time course for early recovering peak. (**F**) Representative UCSC browser track snapshot centered on the promoter of *foraging* showing GAF signal across Nascent CUT&Tag time course for late recovering peak. (**G**) MEME suite motif analysis of early recovering peaks. (**H**) MEME suite motif analysis of late recovering peaks. Merged data of ten biological replicates for control T0, six biological replicates for control T1 and seven biological replicates for control T4.

Overall, 40% of GAF signal is rapidly restored on newly synthesized DNA, and the remainder gradually recovers over 4 hours (Figure 2A). However, at some sites recovery varies: We identified 265 sites that more rapidly acquire GAF signal and 157 sites that recover more slowly (Figure 2B). The 265 early-recovery sites have relatively low signal, but these sites appear maximally occupied immediately after replication fork passage (Figure 2C,E). By contrast, the late-recovery sites are more prominent GAF peaks, but have very low signal on new chromatin, and gradually increase to their maximum after 4 hours (Figure 2D,G). While early recovering peaks have a short GAF consensus motif (Figure 2F), late recovering sites contain longer, more degenerate GAF motifs (Figure 2H), suggesting that motif structure plays a role in the timing of GAF recovery. Early and late recovery peaks also show a substantial difference in peak width, with late recovery peaks showing a median peak width of 1603 basepairs (bp), while early recovery peaks show a median peak width of 942 bp (Figure 2C versus Figure 2D).

We also performed differential analysis on *Drosophila melanogaster* features comprised of promoters, enhancers, and Polycomb response elements (PREs), which are regulatory DNA elements that bring Polycomb group proteins to DNA. We identified 51 (38%) promoters, 41 (30% house-keeping enhancers, 35 (26%) developmental enhancers and 8 (6%) PREs that showed early recovering kinetics. We also identified 167 (49%) promoters, 74 (21%) house-keeping enhancers, 97 (29%) developmental enhancers and (<1%) 2 PREs (Supplementary Figure 1) that showed later recovering kinetics. Early-recovery promoters are enriched for biological function related to the cell cycle, while late-recovery promoters were associated with developmental functions (Table 1). This suggests that slow recovery may be a feature important for regulation of genes active in distinct cell types.

### Chromatin remodeling is required for transcription factor recovery on nascent chromatin

**Figure 3:**
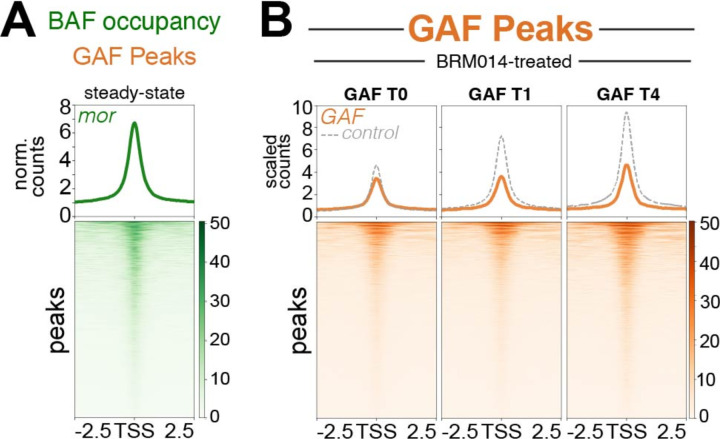
BAF inhibition disrupts GAF rebinding on nascent DNA. (**A**) Heatmap showing CUT&Tag profiling of Moira (BAF component) coverage at all GAF peaks. (**B**) Heatmap aligned to the center of all GAF peaks showing GAF signal over the course of Nascent CUT&Tag experiment treated with 10uM of BRM014. Grey dotted line indicates scaled counts of control data Merged data of two biological replicates for Moira CUT&Tag, four biological replicates for BRM014-treated T0, two biological replicates for BRM014-treated T1 and two biological replicates for BRM014-treated T4.

It is surprising that at some sites transcription factor binding is delayed by hours after passage of the replication fork. Previous studies have suggested that nucleosomes behind the fork may occlude factor binding sites^4,5^. We wondered if chromatin must be remodeled at some genomic sites behind the replication fork to expose factor motifs, and this might delay factor binding. This model implies that chromatin remodeling may be important for chromatin maturation. Specifically, the BAF chromatin remodeler is enriched at promoters that transiently gain nucleosomes that are later repositioned^5^. To assess if BAF is similarly involved at GAF binding sites, we profiled the Moira (BAFp170 homolog) subunit of BAF complexes^16^ using a modified high-yield CUT&Tag protocol^17^. We then plotted Moira coverage at all GAF sites and observed clear Moira enrichment, indicating that BAF associates with GAF-bound sites (Figure 3A).

To directly test if chromatin remodeling is required for GAF binding on nascent chromatin, we inhibited the BAF complex by treating Kc167 cells with BRM014 for 1 hour. BRM014 prevents ATP hydrolysis specifically by the Brahma (Brg1 homolog) catalytic subunit^18^. We then performed Nascent CUT&Tag profiling of GAF. Across all GAF sites on newly replicated chromatin, we observed only a subtle decrease in GAF binding immediately after replication fork passage, but larger decreases from parallel controls 1 hour and 4 hours after replication (Figure 3B). Thus, full restoration of GAF across the genome requires BAF activity.

### BAF regulates transcription factor occupancy on bulk chromatin

**Figure 4.**
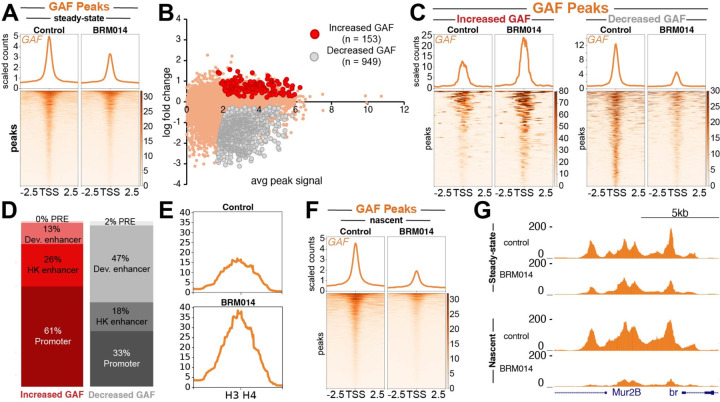
BAF regulates TF occupancy on nascent and bulk chromatin (**A**) Heatmap aligned to the center of all GAF peaks showing CUT&Tag GAF signal in control and 1hr BRM014-treated samples. Read counts are scaled by number of mapped reads. (**B**) MA plot showing fold change in normalized counts comparing control to BRM014-treated samples. Statistical significance determined by two-sided t-test. (**C**) Heatmap aligned to the center of all GAF peaks gaining and losing GAF signal following BRM014-treatment showing GAF signal in control *versus* BRM014-treated samples. (**D**) Bar graph showing proportion of features showing gains and losses in GAF signal following BRM014-treatment. (**E**) GAF coverage at bidirectional promoter element between histones H3 and H4. (**F**) Heatmap aligned to the center of all GAF peaks showing Nascent CUT&Tag GAF signal in control and 1hr BRM014-treated samples. Read counts are scaled by number of mapped reads. (**G**) UCSC browser track snapshot of CUT&Tag and Nascent CUT&Tag experiments centered on the *broad* promoter showing GAF signal for control and BRM014-treated samples.

The loss of GAF binding observed during nascent chromatin maturation suggests that chromatin remodeling might be continually required for factor binding outside of S phase. To investigate how BAF inhibition impacts GAF binding on bulk chromatin, we treated an asynchronous population of Kc167 cells with BRM014 for one hour and profiled GAF binding with standard CUT&Tag. We observed that BRM014 treatment resulted in a ~30% reduction of GAF binding genome-wide (Figure 4A), consistent with the idea that remodeling is continually required for stable GAF binding. Not all sites are equivalently affected: we identified 949 (13%) sites where GAF binding significantly decreases, indicating a strong dependence on chromatin remodeling (Figure 4B,C). Many of the features strongly affected by BRM014 treatment are developmental enhancers (47%), consistent with previous studies^19^ (Figure 4D, Supplementary Figure 2). Interestingly, we also observed 153 sites that show increased GAF signal upon BAF inhibition (Figure 4B,C; Supplementary Figure 2). The majority (61%) of these sites are gene promoters (Figure 4D), such as the H3-H4 bidirectional promoter element (Figure 4E, whose accessibility is normally maintained by chromatin remodelers other than BAF^19^.

In order to compare the effects of BAF inhibition on nascent *versus* bulk chromatin, we next used Nascent CUT&Tag to assess the impact of BAF inhibition on GAF binding to nascent chromatin after 1hr of BRM014 treatment. Globally, we observed that BAF inhibition results in a greater loss of GAF binding on nascent chromatin (Figure 4F,G) when compared to bulk chromatin. Given BAF’s nucleosome eviction activity, these data imply that TF binding sites become occluded by nucleosomes after replication fork passage and require BAF to evict nucleosomes to fully reestablish binding.

### Late recovering sites require BAF activity to reestablish GAF binding on nascent DNA

**Figure 5.**
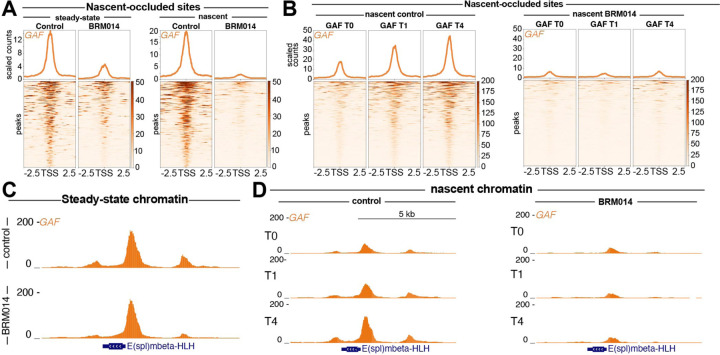
Nascent-occluded peaks are late recovering and highly sensitive to BAF inhibition (**A**) Heatmaps of CUT&Tag and Nascent CUT&Tag data aligned to the center of nascent occluded peaks showing greater loss of GAF signal in Nascent CUT&Tag experiments compared bulk experiments. (**B**) Heatmaps of control and BRM014-treated Nascent CUT&Tag time course data aligned to the center of nascent occluded peaks. (**C**) UCSC browser snapshot of steady state CUT&Tag data showing control and BRM014-treated samples centered on the *Enhancer of split mβ* promoter shown in (**D**) UCSC browser track snapshot centered on the *Enhancer of split mβ* promoter showing GAF signal across Nascent CUT&Tag time course for nascent sensitive.

To better understand differences between bulk and nascent chromatin following BAF inhibition, we compared loss of GAF occupancy in BRM014-treated samples compared to controls. We identified 366 peaks which showed substantially greater loss of GAF binding in nascent BAF inhibition compated to bulk chromatin. On nascent chromatin, these peaks showed near total loss of GAF binding without BAF remodeling (Figure 5A), implying that these sites become nucleosome-occluded following replication fork passage, and that without BAF to evict nucleosomes, GAF is unable to reestablish stable binding. We therefore refer to these sites as nascent occluded sites. We next sought to understand how GAF binding at nascent occluded sites changes over the course of chromatin maturation with and without BAF inhibition. In control conditions, nascent-occluded sites were found to be late recovering peaks, with low levels of GAF occupancy immediately after replication fork passage, but substantial increases in GAF occupancy one hour (T1) and four hours (T4) after replication fork passage (Figure 5B). Conversely, without BAF activity, these sites showed a substantial depletion in GAF occupancy relative to controls immediately after replication fork passage (T0), with no gains in GAF binding during the subsequent stages of chromatin maturation (T1, T4) (Figure 5B). These patterns can be observed at candidate peak regions, such as the promoter of the E(spl)mbeta-HLH (Figure 5C–D). Strikingly, while we saw little change in GAF occupancy at the E(spl)mbeta-HLH promoter in bulk conditions after BAF inhibition (Figure 5C), we observed substantial loss of GAF binding on nascent chromatin (Figure 5D), implying that at certain sites, BAF remodeling is critical for TF binding on nascent chromatin, but dispensable for sustained GAF occupancy on bulk chromatin. Together, these data suggest that nucleosomes deposited after the replication fork antagonize GAF binding, and that chromatin remodeling is essential to enable GAF binding to newly synthesized DNA.

### GAF binding increases at newly replicated GA-rich repeats

**Figure 6.**
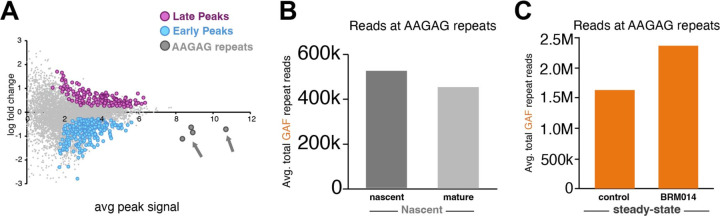
GAF binding increases at newly replicated GA-rich repeats and after BAF inhibition (**A**) MA plot showing fold change T4 *versus* T0 with GA-rich repeats marked (arrows) (**B**) Total average reads at GA-rich repeats in nascent (T0) *versus* mature (T4) chromatin. (**C**) Total average reads at GA-rich repeats in control *versus* BRM014-treated samples. Merged data of four biological replicates.

The 7,000 sites of GAF binding defined by peak calling include a subset of sites with extremely high signal in profiling (Figure 6A). These sites are centered in pericentric heterochromatin on stretches of AAGAG and other GA-rich repeat sequences, an abundant satellite repeat in the Drosophila genome^20^. These satellites have been shown to bind GAF^10^. As GAF coverage at these sites reflects multi-mapped reads across all GA-rich repeats in the genome, we summed these reads to assess GAF binding at satellite blocks. Surprisingly, Nascent CUT&Tag profiles reveal that GAF binding is higher immediately after replication fork passage and then decreases in later timepoints (Figure 6B). Newly replicated chromatin is transiently decondensed, which could provide a window of opportunity for GAF to bind before heterochromatin matures and compacts, thereby displacing GAF. Interestingly, GAF binding also increases substantially at GA-rich repeats following BAF inhibition (Figure 6C). As GAF is lost from many euchromatic sites during BRM014 treatment, GA-rich repeats might be acting as a sink for BAF freed from euchromatin.^21^

## Discussion

Here we present Nascent CUT&Tag, a method for profiling chromatin factors on newly synthesized DNA and quantitatively tracking maturation over time. We demonstrate that Nascent CUT&Tag is effective at profiling a diverse array of chromatin features, including transcription factors such as GAF. As TF binding sites become occluded by nucleosomes following passage of the replication fork, nascent chromatin profiling provides a window into the kinetics and mechanisms by which TFs compete with nucleosomes to reestablish binding. In studying GAF, we find that TF rebinding to nascent chromatin is heterogeneous, with certain sites recovering binding minutes after replication fork passage and other sites requiring hours to fully reestablish binding.

The rates of recovery of distinct chromatin features on nascent chromatin are highly variable, ranging from nearly instantaneous to requiring hours to become fully reestablished^13,14,22^. The deposition of nucleosomes occurs immediately after replication fork passage^23,24^. However, once established on nascent chromatin, it takes time for nucleosomes to fully wrap DNA^25,26^ and reestablish positioning^4^. This process is highly variable between organisms: yeast reestablish nucleosome positioning minutes after replication fork passage^27,28^, while in metazoans, reestablishment of nucleosome positioning^5^ and DNA accessibility^29^ requires over an hour. Interestingly, this recovery is site specific, as house-keeping features show rapid recovery of nucleosome positioning, while sites related to developmental functions show substantial delays^5^. Studies that have assessed TF binding indirectly have shown that TFs likely play a critical role in reestablishing nucleosome positioning on nascent chromatin^30^. However, the molecular mechanisms responsible for helping TFs binding on newly synthesized DNA have remained unclear.

We demonstrate that BAF-mediated chromatin remodeling facilitates full GAF recovery on newly synthesized DNA. As previous studies have shown that TF binding sites become occluded by nucleosomes following passage of the replication fork^5,25^, we propose that BAF activity is essential to evict nucleosomes to allow TFs to reestablish binding at their target sites.

But what distinguishes early recovery sites from late recovery sites? Using Nascent CUT&Tag, we show that early and late recovery sites show differences in GAF motif structure, with early recovery peaks showing short GA-rich motifs and late recovery sites showing longer, more degenerate motifs. Late recovery peaks also show a broad distribution of GAF binding than early recovering sites. As GAF multimerizes and preferentially binds motif arrays relative to single sites^9,31–33^, the broad distribution of GAF at late recovering sites may be due to GAF oligomers bound across extended arrays. Together with our data showing that BAF activity is essential for GAF to fully reestablish binding on nascent chromatin, we propose the following model: After replication fork passage, BAF remodels nucleosomes deposited on new DNA. At early recovery sites, only one or two nucleosomes must be remodeled for GAF to bind a motif. Conversely, at late recovery sites, more extensive remodeling is required to expose the multiple motifs needed for full GAF oligomer binding. As late recovery sites are associated with developmental features, delays in recovery stemming from the need for extended BAF remodeling could aid in regulating selective gene activity required during development. Interestingly, studies that have disrupted TF motifs at developmental features have observed spurious gene activation and disruptions to normal development^34^, emphasizing the importance of motif structure in developmental gene regulation.

The GAF transcription factor is essential for generating open chromatin in cell culture^35^ and early development^36,37^, and at many sites GAF binds to DNA prior to chromatin accessibility^37^. While GAF can bind to reconstituted nucleosomes^33,38^, it possesses little intrinsic chromatin remodeling activity^39^. Instead, GAF appears to recruit chromatin remodelers^40^ to expose factor binding sites and initiate transcription^39,41^. Using a chemical genetics approach to inhibit BAF activity, we demonstrate that BAF-mediated chromatin remodeling is essential for GAF to fully reestablish binding on nascent and bulk chromatin. Given the established role of BAF in moving and evicting nucleosomes, these data imply that at many sites throughout the genome, nucleosomes occupancy is refractory to GAF binding, and that prolonged BAF activity is required for GAF to achieve stable binding.

We observed that certain regions gain GAF binding upon BAF inhibition, including the GA-rich repeats found in pericentromeric heterochromatin, suggesting that these genomic regions could act as a sink for GAF binding following drug treatment^21^. We also observed increased GAF binding at GA-rich repeats immediately after replication fork passage, after which GAF binding decreases at GA-rich repeats as heterochromatin matures and condenses. This pattern is reminiscent of GAF binding at satellites in mitotic cells^15^, where GAF becomes enriched at GA-rich repeats during mitosis, and then redistributes to binding sites in euchromatin during chromatin decondensation in interphase cells. Together, these observations suggest that regardless of the mechanism that liberates GAF from DNA, GA-rich heterochromatic repeats have the potential to act as a sink for unbound GAF, perhaps limiting spurious binding events.

Alternatively, it is possible that GAF binding to newly replicated GA-rich repeats could help to reestablish heterochromatin structure disrupted by replication fork passage, as GAF binding is required to establish repressive heterochromatin at pericentromeric repeats during early *Drosophila* embryogenesis^10^. Interestingly, such a mechanism has also been proposed for the mammalian transcription factor Pax3^42^ which also binds a large repetitive satellite array in human cells, suggesting that TF binding to newly synthesized repeats may be a general mechanism for reestablishing heterochromatin disrupted by replication fork passage. Transcription factor binding sites are prevalent in satellite repeats, and it has been proposed that reiterated arrangement of transcription factor binding sites within repeat sequences is an intrinsic mechanism for heterochromatin formation^43^. In such cases, Nascent CUT&Tag provides a general tool for investigating the timing and mechanisms governing TF binding during heterochromatin replication and chromatin maturation.

## Materials and methods

### Cell culture and drug treatment

Drosophila Kc167 cells (RRID:CVCL_Z834) were grown to log phase in HYQ-SFX Insect medium (Invitrogen) supplemented with 18 mM l-glutamine and harvested as previously described (43). All cell counts and measures of cell size were measured using the Vi-CELL XR Cell Viability Analyzer (www.beckman.com). BRM inhibitor (BRM014) was resuspended to 10 mM in dimethyl sulfoxide and frozen in aliquots. For cell treatments, compounds were added to a cell medium containing 2.0 × 10^6 cells per ml to a final concentration of 10 BRM014 and incubated at room temperature (RT) for times indicated in each experimental paradigm. For nascent timepoints, BRM014 was added immediately before EdU labeling, and remained in media for the duration of the EdU pulse. For chase timepoints, BRM014 was included in all media used to wash and resuspend cells, meaning that chases of 1hr and 4hrs reflect treatment times of BRM014 of 1hr and 4hrs respectively. For steady state experiments, compounds were added to a cell medium containing 2.0 × 10^6 Kc167 cells per ml to a final concentration of 10 µM BRM014 and incubated at room temperature (RT) for times indicated in each experimental paradigm. Aliquots of 500K cells were taken for bulk chromatin CUT&Tag profiling

### Nascent CUT&Tag

Kc167 were grown to confluence and split down to a concentration of one million Drosophila Kc167 cells per ml 20 hours before the start of EdU labelling. EdU analogue was then added to the media at a concentration of 10uM and incubated for 15 minutes. Cells were then spun down and resuspended in 30mL cell culture media containing 20uM cold thymidine. Cells were spun down again and resuspended in cell culture chase media (equal parts fresh media and conditioned media + 20uM thymidine). A volume was taken corresponding to 2 million cells per ml at current cell density and placed on ice as the nascent timepoint sample. The remainder of the cells were left to chase for timepoints specified. Additional volumes of samples were taken 1hr post EdU addition and 4 hours post EdU addition. Once all samples were taken, CUT&Tag with phenol-chloroform DNA extraction was performed as described^6,44–46^. Following extraction, DNA was solubilized in 10uL of H2O. Click chemistry was then performed to conjugate biotin onto replicated DNA molecules. Following completion of click-chemistry, SPRI bead cleanup was performed and DNA was resuspended in 20uls of water. Replicated DNA was then pulled down using Streptavidin T1 beads according to manufacturer’s instructions. Following completion of pull-down 21 µL of DNA bound to streptavidin T1 beads was mixed with a universal i5 and a uniquely barcoded i7 primer and amplified with NEB Q5 high-fidelity 2× master mix (catalog no. M0492S). The libraries were purified with 1.1× volume of Sera-Mag carboxylate-modified magnetic beads and subjected to LabChip DNA analysis and Illumina sequencing. Barcoded Nascent CUT&Tag libraries were pooled at equal volumes to normalize read counts between samples.

### CUT&Tag data processing and analysis

Libraries were sequenced on an Illumina NovaSeq instrument with paired-end 50 × 50 reads Barcoded CUTAC libraries were pooled at equal volumes within group to allow for normalization of total reads across samples.

Adapters were clipped by cutadapt (http://dx.doi.org/10.14806/ej.17.1.200) version 2.9 with the following parameters:

-j 8 --nextseq-trim 20 -m 20 -a AGATCGGAAGAGCACACGTCTGAACTCCAGTCA -AAGATCGGAAGAGCGTCGTGTAGGGAAAGAGTGT -Z. Clipped reads were aligned by Bowtie2^47^ to the UCSC D. melanogaster Dm6 reference sequence^48^ with the following parameters:

--very-sensitive-local --soft-clipped-unmapped-tlen --dovetail --no-mixed --no-discordant -q -- phred33 -I 10 -X 1000. Clipped reads were also aligned by Bowtie2^47^ to the UCSC D. melanogaster Dm6 reference sequence^48^ with the following parameters: --end-to-end --very-sensitive --no-overlap --no-dovetail --no-mixed --no-discordant -q --phred33 -I 10 -X 1000. Properly paired reads were extracted from the alignments by SAMTools (version 1.9)^49^. Normalized count tracks in bigwig format were also made by bedtools^50^ 2.30.0 genomecov which are the fraction of counts at each base pair scaled by the size of the reference sequence (137,567,484) so that if, the scaled counts were uniformly distributed, there would be 1 at each position. Bigwig files were then uploaded to Galaxy^51^ and heatmaps were generated using the computematrix function in deepTools (version 3.5.1)^52^.

A bedfile containing a list of all D. melanogaster promoters, enhancers and PREs was used by computematrix function to compare scaled normalized reads from bedgraph files aligned at midpoint all D. melanogaster promoters, enahancers and PREs. The output of the computematrix function was visualized using the plotHeatmap function in Galaxy.

To generate plots showing changes in the GAF coverage in control versus BRM014-treated cells in nascent vs. steady state experiments, the total GAF signal at each peak promoter from 500-bp upstream to 500-bp downstream was summed using the multiBigwigSummary function from Galaxy deepTools.

### Generating scaling factors

To generate a scaling factor to normalize read counts between nascent timepoints, we divided the total mapped reads of each sample in the experiment by the average number of reads at the nascent timepoint. We generated scaling values for each timepoint across all experiments. We then averaged scaling values at each timepoint across experiments to generate the averaged scaling value for that timepoint. We used the averaged scaling value in the computeMatrix function to scale the normalized read counts for each timepoint accordingly.

To generate a scaling factor to normalize read counts between control and BRM014-treated samples, we divided the total mapped reads in each paired BRM014-treated and control sample and averaged the values. We used the averaged scaling value in the computeMatrix function to scale the normalized read counts for each timepoint accordingly.

### Identifying nascent sensitive peaks

We identified nascent sensitive peaks first by summing total signal at each peak in control and BRM014 treated conditions in steady state and nascent experiments. We then filtered our lists to contain only peaks that showed statistically significant differences between control and drug treated conditions using two-sided t-tests. We then calculated the absolute difference by subtracting the total signal in control conditions by BRM014-treated conditions for steady state and nascent samples. We then sorted peaks by those which showed losses greater than 5 arbitrary units in the nascent condition when compared to the steady state condition. Five was chosen, as this was the approximate inflection point in a knee plot of all differences between nascent and bulk chromatin. We also generated a fold change value by dividing the signal in control groups by signal in BRM014-treated groups. We then compared those ratios between nascent and bulk chromatin experiments. Peaks in which the fold change in nascent samples was 1.5-fold greater than in bulk chromatin experiments we deemed to be sensitive in nascent. The 1.5-fold threshold was selected as this was the approximate inflection point in a knee plot of nascent *versus* bulk chromatin ratios. Peaks identified by raw difference and fold change were merged and duplicates removed to generate the final nascent-sensitive list of peaks.

## Supplementary Material

Supplement 1

Supplement 2

## Data Availability

All data needed to evaluate the conclusions in the paper are present in the paper and/or the Supplementary Materials. All primary sequencing data have been deposited in the NCBI’s Gene Expression Omnibus (GEO) under accession code GSE*****.
