# Supplementary material for: Nascent CUT&Tag captures transcription factor binding after chromatin duplication
Source: bioRxiv. 2025 Oct 15:2025.10.13.682212. Preprint. [Version 1] doi: 10.1101/2025.10.13.682212 (PMC12632889; doi:10.1101/2025.10.13.682212)
Supplement: Supplement 2 [file NIHPP2025.10.13.682212v1-supplement-2.pdf]

bioRxiv preprint doi: https://doi.org/10.1101/2025.10.13.682212; this version posted October 15, 2025. The copyright holder for this preprint (which was not certified by peer review) is the author/funder, who has granted bioRxiv a license to display the preprint in perpetuity. It is made available under aCC-BY 4.0 International license.

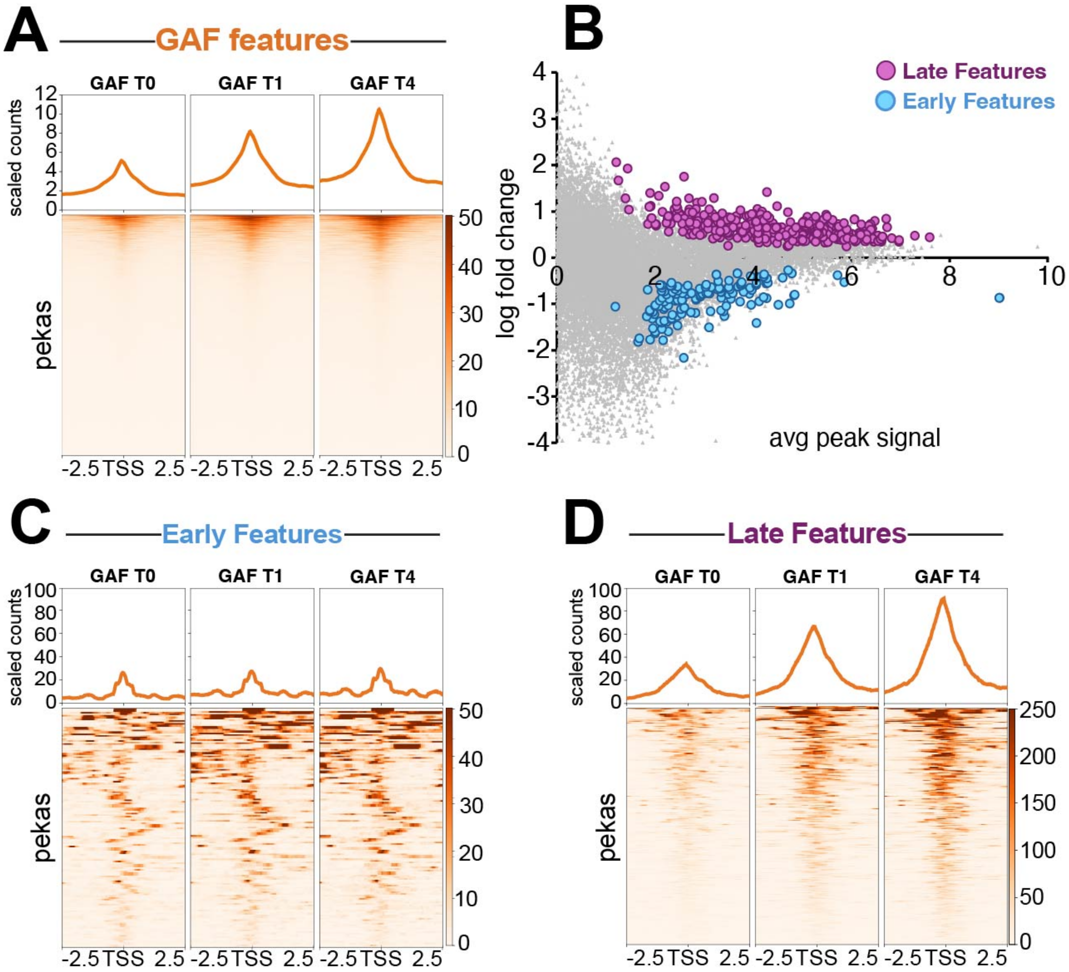

Supplementary Figure 1. GAGA factor is lost on nascent chromatin and recovers over time
(**A**) Heatmap aligned to the center of all *Drosophila* features showing GAF signal over the course of Nascent CUT&Tag experiment. Read counts are scaled by number of mapped reads.((**B**) MA plot showing fold change in normalized counts comparing T4 to T0. Statistical significance determined by two-sided t-test. (**C**) Heatmap aligned to the center of all early recovering GAF features showing GAF signal over the course of Nascent CUT&Tag experiment (**D**) Heatmap aligned to the center of late recovering GAF features showing GAF signal over the course of Nascent CUT&Tag experiment.

bioRxiv preprint doi: https://doi.org/10.1101/2025.10.13.682212; this version posted October 15, 2025. The copyright holder for this preprint (which was not certified by peer review) is the author/funder, who has granted bioRxiv a license to display the preprint in perpetuity. It is made available under aCC-BY 4.0 International license.

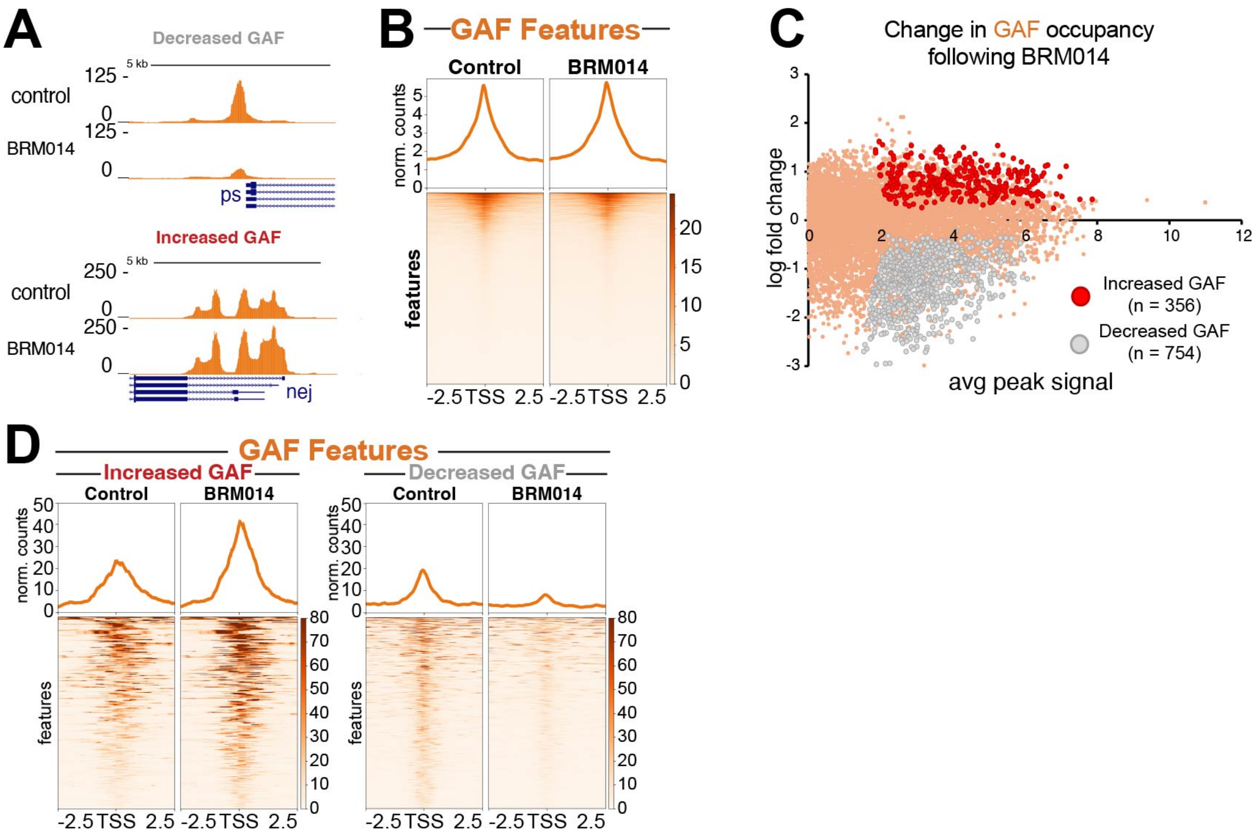

Supplementary Figure 2. BAF inhibition alters GAF binding on bulk chromatin
(**A**) Representative UCSC browser track snapshot of control and BRM014-treated samples showing GAF signal at peaks gaining and losing GAF signal following BRM014-treatment. (**B**) Heatmap aligned to the center of all GAF features showing CUT&Tag GAF signal in control and 1hr BRM014-treated samples. Read counts are scaled by number of mapped reads (**C**) MA plot showing fold change in normalized counts at features comparing control to BRM014-treated samples. Statistical significance determined by two-sided t-test. (**D**) Heatmap aligned to the center of all GAF features gaining and losing GAF signal following BRM014-treatment showing GAF signal in control *versus* BRM014-treated samples.
